# Criminal Abortion

**Published:** 1859-01

**Authors:** 


CRIMINAL ABORTION.—We would call the attention of the profession to the queries appended to Dr. Storer's article on this subject in the present number of this Journal, and solicit their co-operation with the author in the furtherance of his inquiries.

